# Bayesian and Frequentist Inferences on a Type I Half-Logistic Odd Weibull Generator with Applications in Engineering

**DOI:** 10.3390/e23040446

**Published:** 2021-04-10

**Authors:** Mahmoud EL-Morshedy, Fahad Sameer Alshammari, Abhishek Tyagi, Iberahim Elbatal, Yasser S. Hamed, Mohamed S. Eliwa

**Affiliations:** 1 Department of Mathematics, College of Science and Humanities in Al-Kharj, Prince Sattam bin Abdulaziz University, Al-Kharj 11942, Saudi Arabia; f.alshammari@psau.edu.sa; 2 Department of Mathematics, Faculty of Science, Mansoura University, Mansoura 35516, Egypt; mseliwa@mans.edu.eg; 3 Department of Statistics, Chaudhary Charan Singh University, Meerut 250004, India; abhishektyagi033@gmail.com; 4 Department of Mathematics and Statistics, College of Science, Imam Mohammad Ibn Saud Islamic University (IMSIU), Riyadh 13318, Saudi Arabia; iielbatal@imamu.edu.sa; 5 Department of Mathematics and Statistics, College of Science, Taif University, Taif 21944, Saudi Arabia; yasersalah@tu.edu.sa

**Keywords:** odd Weibull-G family, Type I half logistic distribution, estimation methods, hazard rate function, simulation

## Abstract

In this article, we have proposed a new generalization of the odd Weibull-G family by consolidating two notable families of distributions. We have derived various mathematical properties of the proposed family, including quantile function, skewness, kurtosis, moments, incomplete moments, mean deviation, Bonferroni and Lorenz curves, probability weighted moments, moments of (reversed) residual lifetime, entropy and order statistics. After producing the general class, two of the corresponding parametric statistical models are outlined. The hazard rate function of the sub-models can take a variety of shapes such as increasing, decreasing, unimodal, and Bathtub shaped, for different values of the parameters. Furthermore, the sub-models of the introduced family are also capable of modelling symmetric and skewed data. The parameter estimation of the special models are discussed by numerous methods, namely, the maximum likelihood, simple least squares, weighted least squares, Cramér-von Mises, and Bayesian estimation. Under the Bayesian framework, we have used informative and non-informative priors to obtain Bayes estimates of unknown parameters with the squared error and generalized entropy loss functions. An extensive Monte Carlo simulation is conducted to assess the effectiveness of these estimation techniques. The applicability of two sub-models of the proposed family is illustrated by means of two real data sets.

## 1. Introduction

In the last few decades, many efforts have been made to improve the modelling of different types of data arising from several fields such as actuarial, environmental, economics, engineering, medicine and biological sciences. These activities have led to the creation of powerful methods for creating new lifetime models. Among these various techniques of generating new models, in the widely used method, we define new classes of univariate continuous models by adding one or more additional shape parameter(s) to the baseline model. This induction of additional parameter(s) has been proved useful in studying the tail properties and also for providing wider modelling flexibility of the generator (G) family. Thus, several classes by adding one or more parameters have been proposed in the statistical literature. Initially, Eugene et al. [1] introduced a general class of distributions from the logit of the beta random variable. They studied beta-normal distribution as a special model of this family and showed that it is enough capable of modelling the symmetric heavy-tailed, skewed and bimodal data. With a similar idea as of Eugene et al. [1], Zografos and Balakrishnan [2] developed the gamma-G family by Stacy’s generalized gamma variables and discussed its important properties.

Cordeiro and Castro [3] introduced Kumaraswamy-G (Kw-G) family based on the Kumaraswamy distribution. They investigated some special distributions of this family such as the Kw-Normal, Kw-Weibull, Kw-gamma, Kw-Gumbel and Kw-inverse Gaussian distribution. Tahir et al. [4] developed logistic-X family based on a logistic random variable. They figured out that its density function can be symmetrical, left-skewed, right-skewed, and reversed-J shaped, and can take increasing, decreasing, bathtub, and upside-down bathtub shaped hazard rates. They also examined logistic-Fréchet distribution as a particular model. Tahir et al. [5] introduced a new generator from the Weibull random variable called the Weibull-G family. They showed that the density and hazard rate function can assume a variety of shapes. Cordeiro et al. [6] invented a generalized odd log-logistic family of distributions with two extra shape parameters. They also produced the log-odd log-logistic Weibull regression model with censored data based on the odd log-logistic-Weibull distribution. Afify et al. [7] formulated odd exponentiated half logistic-G family. They provided some special models of this family and demonstrated that special models are capable of modelling various shapes of ageing and failure criteria.

Haghbin et al. [8] introduced a new generalized odd log-logistic family of distributions with three extra parameters. The authors pointed out that the family contains several important classes available in the literature as sub-models such as the proportional reversed hazard rate and odd log-logistic classes. El-Sherpieny and EL-Sehetry [9] developed Kumaraswamy type I half logistic-G family and figured out that the new family extends well-known distributions as well as it provides great flexibility to model specific real data. El-Morshedy and Eliwa [10] originated a new generator of continuous distributions called the odd flexible Weibull-H family and studied its properties.

Recently, Eliwa et al. [11] presented the odd Chen-G family and discussed its various properties. Further, Eliwa et al. [12] provided an exponentiated version of the odd Chen-G family and named it as Exponentiated odd Chen-G family. They also pointed out that its hazard rate function can be increasing, decreasing, unimodal-bathtub shaped. Alizadeh et al. [13] proposed a new class of distributions called the odd log-logistic Lindley-G family. The authors also demonstrated a special model of the proposed family that can have symmetrical, right-skewed, left-skewed and reversed-J shaped densities, and decreasing, increasing, bathtub, unimodal and reversed-J shaped hazard rates. Badr et al. [14] introduced a new family of flexible distributions called the transmuted odd Fréchet-G family and they exhibited that the particular models of the proposed family can assume different kinds of monotonic and non-monotonic shapes for the probability density and hazard rate functions. Tahir et al. [15] proposed a new Kumaraswamy-G family of distributions for the bounded unit interval. The authors also studied the new Kumaraswamy-Weibull (NKwW) distribution as a special model of the Kumaraswamy-G family. Eliwa et al. [16] introduced a discrete version of the Gompertz-G family. They also examined some of its distributional and reliability properties and discussed three special models in detail. Most recently, Zaidi et al. [17] invented a new class named as log-logistic tan generalized family. They also showed that the special models of this family can assume a variety of shapes for density and hazard function.

Bourguignon et al. [18] invented a new continuous family of models, which became popular as the odd Weibull-G (OW-G) family. The cumulative distribution function (CDF) and the probability density function (PDF) of the OW-G family, respectively, can be written as

(1) $$\Pi_{\mathrm{OW}\text{-}G}\left(x;\beta ,\varphi \right)=1-e^{-{\left(\frac{G(x;\varphi )}{\bar{G}\left(x;\varphi \right)}\right)}^{\beta }};x>0$$

and

(2) $$\pi_{\mathrm{OW}\text{-}G}\left(x;\beta ,\varphi \right)=\beta g\left(x;\varphi \right)\frac{G{\left(x;\varphi \right)}^{\beta -1}}{\bar{G}{\left(x;\varphi \right)}^{\beta +1}}e^{-{\left(\frac{G(x;\varphi )}{\bar{G}\left(x;\varphi \right)}\right)}^{\beta }};x>0,$$

where the shape parameter $\beta \in {ℜ}^{+}$ (positive real line), g(x;**φ**) and $\bar{G}(x;$**φ**)=1−G(x;**φ**) are, respectively, the PDF and survival function of the baseline model depend on a vector of parameters, φ. Cordeiro et al. [19] provided a new flexible generator of models with an additional shape parameter $\lambda \in {ℜ}^{+}$, and labeled it as type I half logistic-G (TIHL-G) family. The CDF and PDF of the TIHL-G family, respectively, can be written as

(3) $$\Pi_{\mathrm{TIHL}\text{-}G}^{*}\left(x;\lambda ,\varphi \right)=\frac{1-{\left[1-G(x;\varphi )\right]}^{\lambda }}{1+{\left[1-G(x;\varphi )\right]}^{\lambda }};x>0$$

and

(4) $$\pi_{\mathrm{TIHL}\text{-}G}^{*}\left(x;\lambda ,\varphi \right)=\frac{2\lambda g\left(x;\varphi \right){\left[1-G(x;\varphi )\right]}^{\lambda -1}}{{\left[1+{\left[1-G(x;\varphi )\right]}^{\lambda }\right]}^{2}};x>0.$$

In this article, the main objective is to construct a new class of the type I half logistic odd Weibull-G (Type I HLOW-G) by combining the type I half logistic and odd Weibull families. The CDF of the Type I HLOW-G family is

(5) $$F_{\mathrm{Type}\;I\;\mathrm{HLOW}\text{-}G}\left(x;\lambda ,\beta ,\varphi \right)=\left[1-e^{-\lambda {\left(\frac{G(x;\varphi )}{\bar{G}\left(x;\varphi \right)}\right)}^{\beta }}\right]{\left[1+e^{-\lambda {\left(\frac{G(x;\varphi )}{\bar{G}\left(x;\varphi \right)}\right)}^{\beta }}\right]}^{-1};x>0,$$

where $\lambda \in {ℜ}^{+}$ and $\beta \in {ℜ}^{+}$ are the scale and shape parameter, respectively. The odds ratio $\frac{G(x;\varphi )}{\bar{G}\left(x;\varphi \right)}$ satisfies the following conditions: $\frac{G(x;\varphi )}{\bar{G}\left(x;\varphi \right)}$∈[l,m] for 0<l<m<∞; $\frac{G(x;\varphi )}{\bar{G}\left(x;\varphi \right)}$ is monotonically non-decreasing and differentiable, and $\frac{G(x;\varphi )}{\bar{G}\left(x;\varphi \right)}$→ *l* as x→0, but $\frac{G(x;\varphi )}{\bar{G}\left(x;\varphi \right)}$→ m as x→∞. For further details regarding the physical meaning of the odds ratio, see Cooray [20]. The PDF of Type I HLOW-G family can be inscribed as

(6) $$f_{\mathrm{Type}\;I\;\mathrm{HLOW}\text{-}G}\left(x,\lambda ,\beta ,\varphi \right)=\frac{2\lambda \beta g\left(x;\varphi \right)G{\left(x;\varphi \right)}^{\beta -1}}{\bar{G}{\left(x;\varphi \right)}^{\beta +1}}{\left[1+e^{-\lambda {\left(\frac{G(x;\varphi )}{\bar{G}\left(x;\varphi \right)}\right)}^{\beta }}\right]}^{-2}e^{-\lambda {\left(\frac{G(x;\varphi )}{\bar{G}\left(x;\varphi \right)}\right)}^{\beta }};x>0.$$

The hazard (failure) rate function (HRF) of the Type I HLOW-G family is

(7) $$h_{\mathrm{Type}\;I\;\mathrm{HLOW}\text{-}G}\;\left(x,\lambda ,\beta ,\varphi \right)=\frac{\lambda \beta g\left(x;\varphi \right)G{\left(x;\varphi \right)}^{\beta -1}}{\bar{G}{\left(x;\varphi \right)}^{\beta +1}}{\left[1+e^{-\lambda {\left(\frac{G(x;\varphi )}{\bar{G}\left(x;\varphi \right)}\right)}^{\beta }}\right]}^{-1};x>0.$$

The main objectives of proposing the Type I HLOW-G family can be stated as follows: to list the special distributions containing different shaped HRF (increasing, decreasing, unimodal, and Bathtub); to provide more flexibility for skewness and kurtosis as compared to the baseline model; to consistently produce superior fits as compared to other generated distributions with the same baseline model; and to illustrate how different estimators of the unknown parameters of the particular sub-model perform for varied sample size and various combinations of the parametric values. Table 1 reports G(x;φ)/$\bar{G}\left(x;\varphi \right)$ with the associated parameters for some particular models.

The rest of the study is organized as follows: In Section 2, linear representation for the Type I HLOW-G density are derived. Section 3 contains several statistical characteristics of the Type I HLOW-G family. In Section 4, some particular distributions are investigated. In Section 5, the estimation of family parameters are explored through different estimation techniques, viz, method of maximum likelihood, least squares and weighted least squares methods, Cramér-von Mises distance estimators, and Bayesian estimation. Section 6 deals with the Monte Carlo simulations for small, moderate, and large samples. In Section 7, two real life data sets are examined to exemplify the flexibility of the Type I HLOW-G family. Finally, some conclusions about the proposed study are made in Section 8.

## 2. Linear Representation for the Type I HLOW-G Density

In this Section, we have used some useful expansion functions that follow the generalized binomial series. If ∣s∣<1 and d>0 is a real non-integer, then the following power series holds

(8) (1+s)−d=∑l=0∞−dlsl

and

(9) (1−s)−d=∑l=0∞(−1)l−dlsl.

Using Equations (8) and (9) to the last term in Equation (6), we get

(10) fTypeIHLOW-G(x,λ,β,φ)=2λβg(x;φ)∑i=0∞−2iG(x;φ)β−1G¯(x;φ)β+1e−λ(i+1)G(x;φ)G¯(x;φ)β,

expanding $e^{-\lambda \left(i+1\right){\left(\frac{G(x;\varphi )}{\bar{G}\left(x;\varphi \right)}\right)}^{\beta }}$ through exponential series, we can write

$$e^{-\lambda \left(i+1\right){\left(\frac{G(x;\varphi )}{\bar{G}\left(x;\varphi \right)}\right)}^{\beta }}=\sum_{j=0}^{\infty }\frac{{\left(-1\right)}^{j}\lambda^{j}{\left(i+1\right)}^{j}}{j!}\frac{G{\left(x;\varphi \right)}^{\beta j}}{\bar{G}{\left(x;\varphi \right)}^{\beta j}},$$

insert the above term in Equation (10) we get

(11) fTypeIHLOW-G(x,λ,β,φ)=2λβ∑i,j=0∞(−1)jλj(i+1)jj!−2ig(x;φ)G(x;φ)β(j+1)−1G¯(x;φ)β(j+1)+1,

by using Equation (9) into Equation (11), we get

(12) G¯(x;φ)−β(j+1)+1=∑k=0∞(−1)k−β(j+1)−1kG(x;φ)k,

inserting Equation (12) in Equation (11), the Type I HLOW-G density function can be written as infinite mixture of Expo-G density function as follows

(13) $$f_{\mathrm{Type}\;I\;\mathrm{HLOW}\text{-}G}\left(x,\lambda ,\beta ,\varphi \right)=\sum_{j,k=0}^{\infty }\varkappa_{{}_{\left(j,k\right)}}^{\left(\lambda ,\beta \right)}\;\pi_{\left[\beta (j+1)+k\right]}\left(x;\varphi \right),$$

where

ϰ(j,k)(λ,β)=2β∑i=0∞(−1)j+kλj+1(i+1)jj!(β(j+1)+k)−2i−β(j+1)−1k

and

$$\pi_{\left[\beta (j+1)+k\right]}\left(x;\varphi \right)=\left[\beta (j+1)+k\right]g\left(x;\varphi \right)G^{\left[\beta (j+1)+k\right]-1}\left(x;\varphi \right),$$

is the Exp-G PDF with power parameter $\left[\beta (j+1)+k\right]$. As Type I HLOW-G density function can be expressed as an infinite linear combination of Exp-G density functions, therefore several mathematical quantities of the Type I HLOW-G family can be determined obviously from those of Exp-G family. For instance, the simple and incomplete moments, probability weighted moments, moment generating function of the Type I HLOW-G model can be derived directly from those expressions of the Exp-G model.

Similarly, the CDF of the Type I HLOW-G family can also be written as a mixture of Exp-G CDF as follows

(14) $$F_{\mathrm{Type}\;I\;\mathrm{HLOW}\text{-}G}\left(x;\lambda ,\beta ,\varphi \right)=\sum_{j,k=0}^{\infty }\varkappa_{{}_{\left(j,k\right)}}^{\left(\lambda ,\beta \right)}\;\Pi_{\left[\beta (j+1)+k\right]}\left(x;\varphi \right),$$

where $\Pi_{\left[\beta (j+1)+k\right]}$ is the Exp-G CDF with power parameter $\left[\beta (j+1)+k\right]$. The expressions in Equations (13) and (14) are the main result of this segment.

## 3. Statistical Properties

### 3.1. Quantile Function (QF)

The QF plays a very crucial role not only in theoretical aspect but also in various statistical applications and Monte Carlo methods. Monte Carlo simulations use QF to simulate continuous random variables from existing and newly proposed models. The QF of the Type I HLOW-G family can be derived by inverting Type I HLOW-G CDF as follows

$$F_{\mathrm{Type}\;I\;\mathrm{HLOW}\text{-}G}^{-1}\left(u\right)=Q\left(u\right)=G^{-1}\left[\frac{{\left\{-\frac{1}{\lambda }\log\left[\frac{1-u}{1+u}\right]\right\}}^{\frac{1}{\beta }}}{1+{\left\{-\frac{1}{\lambda }\log\left[\frac{1-u}{1+u}\right]\right\}}^{\frac{1}{\beta }}}\right],$$

where Q(u) denotes the QF corresponding to G(x;φ), $G^{-1}\left(.\right)$ is the inverse of the baseline CDF, and *U* is a uniform random variable on (0,1). Subject to the QF, Kenney and Keeping [21] and Moors [22] proposed two of the earliest skewness (Sk) and kurtosis (Ku) measures, respectively, as follows

$$Sk=\frac{Q\left(\frac{3}{4}\right)+Q\left(\frac{1}{4}\right)-2Q\left(\frac{1}{2}\right)}{Q\left(\frac{3}{4}\right)-Q\left(\frac{1}{4}\right)}\;\;\mathrm{and}\;\;Ku=\frac{Q\left(\frac{7}{8}\right)-Q\left(\frac{5}{8}\right)+Q\left(\frac{3}{8}\right)-Q\left(\frac{1}{8}\right)}{Q\left(\frac{6}{8}\right)-Q\left(\frac{2}{8}\right)}.$$

One remarkable thing about these measures is that they are less affected by outliers and they can be obtained even for distributions without moments.

### 3.2. Moments and Moment Generating Function (MGF)

Various important features such as Sk, Ku, measures of central tendency and variation can be derived from the *r*th moment. The *r*th moment of the Type I HLOW-G family can be developed by Equation (13) as follows

(15) $$\mu_{r}^{{}^{/}}=E\left(X^{\;r}\right)=\sum_{j,k=0}^{\infty }\varkappa_{{}_{\left(j,k\right)}}^{\left(\lambda ,\beta \right)}\;E\left(W_{\left[\beta (j+1)+k\right]}^{\;r}\right),$$

where $W_{\left[\beta (j+1)+k\right]}^{\;r}$ denotes the Exp-G random variable (RV) with power parameter $\left[\beta (j+1)+k\right]$. For ξ>0, the *r*th moment of the Exp-G RV can be proposed by

$$E\left(W_{\xi }^{r}\right)=\xi \int_{-\infty }^{\infty }x^{r}g\left(x;\varphi \right)G{\left(x;\varphi \right)}^{\xi -1}dx\;\;\mathrm{or}\;\;E\left(W_{\xi }^{r}\right)=\xi \int_{0}^{1}u^{\xi -1}Q_{G}{\left(u\right)}^{r}du,$$

where $Q_{G}\left(u\right)=G^{-1}\left(u\right)$. The Sk and the Ku can be calculated, respectively, as follows

$$Sk=\left(\mu_{3}^{{}^{\prime }}-3\mu_{2}^{{}^{\prime }}\mu_{1}^{{}^{\prime }}+\mu_{1}^{{}^{\prime }3}\right)/{\left(\mu_{2}^{{}^{\prime }}-\mu_{1}^{{}^{\prime }2}\right)}^{3/2}$$

and

$$Ku=\left(\mu_{4}^{{}^{\prime }}-4\mu_{3}^{{}^{\prime }}\mu_{1}^{{}^{\prime }}-3\mu_{2}^{{}^{\prime }2}+12\mu_{2}^{{}^{\prime }}\mu_{1}^{{}^{\prime }2}-6\mu_{1}^{{}^{\prime }4}\right)/{\left(\mu_{2}^{{}^{\prime }}-\mu_{1}^{{}^{\prime }2}\right)}^{2}.$$

If the RV *X* has the Type I HLOW-G family, then the *n*th central moments and MGF can be derived, respectively, as

Mn(x)=∑r=0∞∑j,k=0∞nr(−μ1/)n−rϰ(j,k)(λ,β)E(Wβ(j+1)+kr)

and

(16) $$M_{X}\left(t\right)=E\left(e^{tX}\right)=\sum_{j,k=0}^{\infty }\varkappa_{{}_{\left(j,k\right)}}^{\left(\lambda ,\beta \right)}\;M_{\left[\beta (j+1)+k\right]}\left(t\right),$$

here $M_{\left(\beta (j+1)+k\right)}\left(t\right)$ is the MGF of the Exp-G family. A second alternative formula to the MGF of the Type I HLOW-G family is based of the QF, and can be presented as

(17) $$M_{X}\left(t\right)=\sum_{j,k=0}^{\infty }\varkappa_{{}_{\left(j,k\right)}}^{\left(\lambda ,\beta \right)}\upsilon \left(t,\left[\beta (j+1)+k\right]\right),$$

where $\upsilon \left(t,\kappa \right)=\kappa \int_{0}^{1}u^{\kappa -1}e^{tQ_{G}\left(u\right)}du$.

### 3.3. Incomplete Moments and Mean Deviation

The incomplete moments are mostly used to describe the Bonferroni and Lorenz curves (BLC) which are greatly applicable in reliability, insurance, medicine, economics, and demography. Moreover, the BLC can be utilized to evaluate the mean deviations (MDs). For more detail on BLC, one can refer Bonferroni [23] and Lorenz [24]. Let X follows the Type I HLOW-G family, the *s*th incomplete moments can be derived as

(18) $$\eta_{s}\left(t\right)=\sum_{j,k=0}^{\infty }\varkappa_{{}_{\left(j,k\right)}}^{\left(\lambda ,\beta \right)}\;\Upsilon_{\left[\beta (j+1)+k\right]}^{s}\left(t\right);s>0,$$

where $\eta_{s}\left(t\right)=\int_{-\infty }^{t}x^{s}f_{\mathrm{Type}\;I\;\mathrm{HLOW}\text{-}G}\left(x,\lambda ,\beta ,\varphi \right)dx$ and $\Upsilon_{\left[\beta (j+1)+k\right]}^{s}\left(t\right)$ represents the *s*th incomplete moments of the Exp-G family. Based on Equations (15) and (18), the MDs of the Type I HLOW-G family about the mean ($\mu_{1}^{{}^{/}}$) and median can be listed, respectively, as

(19) $$\delta_{\mu_{1}^{{}^{/}}}\left(X\right)=E|X-\mu_{1}^{{}^{/}}|=2\mu_{1}^{{}^{/}}F_{\mathrm{Type}\;I\;\mathrm{HLOW}\text{-}G}\left(\mu_{1}^{{}^{/}}\right)-2\eta_{1}\left(\mu_{1}^{{}^{/}}\right)$$

and

(20) $$\delta_{Q\left(\frac{1}{2}\right)}\left(X\right)=E|X-Q\left(\frac{1}{2}\right)|=\mu_{1}^{{}^{/}}-2\eta_{1}\left[Q\left(\frac{1}{2}\right)\right].$$

The MD is the most appropriate tool to measure the average absolute deviation of the observations. It is used in many important fields including economics, insurance and reliability theory.

For a positive RV, the BLC of the Type I HLOW-G family for a given probability *p* can be written as

(21) $$\mathrm{Bonferroni}\left(p\right)=\frac{1}{p\mu_{1}^{{}^{/}}}\eta_{1}\left(q\right)\;\;\mathrm{and}\;\;\mathrm{Lorenz}\left(p\right)=\frac{1}{\mu_{1}^{{}^{/}}}\eta_{1}\left(q\right),$$

where q=Q(p) is the QF of the Type I HLOW-G family at *p*.

### 3.4. Probability Weighted Moments

The probability weighted moments (PWMs) are the generalization of the ordinary moments of a probability distribution (PD) and can be obtained for any PD whose usual moments exist. The PWM method can usually be applied to estimate the parameters of a PD whose inverse CDF cannot be extracted in a closed-form. If the RV *X* has the Type I HLOW-G family, then the (r,s)th PWM is given as

(22) $$\Xi_{\left(r,s\right)}=E\left\{X^{r}F{\left(x\right)}^{s}\right\}=\sum_{j,k=0}^{\infty }\epsilon_{j,k}^{\left(s\right)}\;\Psi_{\left[\beta (j+1)+k\right]}^{s},$$

where $\Psi_{\left[\beta (j+1)+k\right]}^{s}$ is the PWM of the Exp-G family with power parameter [β(j+1)+k], and

ϵj,k(s)=2β∑i,m=0∞(−1)m+j+kλj+1(i+m)jj!(β(j+1)+k)sm−s−2i−β(j+1)−1k.

### 3.5. Moments of Residual and Reversed Residual Lifetimes

In reliability analysis, the additional lifetime provided that a unit has survived until time *t* is known as the residual lifetime (RLT) function of the unit, and it is presented by the conditional RV X−x|X>x. If the RV *X* has the Type I HLOW-G family, the *r*th order moment of the RLT can be obtained as

(23) $$\varpi_{r}\left(x\right)=E\left[{\left(X-x\right)}^{r}|X>x\right]=\frac{1}{1-F_{\mathrm{Type}\;I\;\mathrm{HLOW}\text{-}G}\left(x,\lambda ,\beta ,\varphi \right)}\sum_{j,k=0}^{\infty }\varkappa_{{}_{j,k}}^{*}\Psi_{\left[\beta (j+1)+k\right]}^{*r}\left(x\right),$$

where $\varkappa_{{}_{j,k}}^{*}=\varkappa_{{}_{\left(j,k\right)}}^{\left(\lambda ,\beta \right)}$ ∑m=0rrm(−t)r−m, and $\Psi_{\left[\beta (j+1)+k\right]}^{*r}$$\left(x\right)=\int_{x}^{\infty }y^{r}$$\pi_{\left[\beta (j+1)+k\right]}\left(y\right)dy$. Putting r=1 in Equation (23), the mean RLT (MRLT) can be derived. The MRLT is a well-known concept in reliability and survival analysis (see, Gupta and Gupta [25]; Kundu and Nanda [26]).

The reversed mean residual lifetime (RRLT) (or inactivity time) can be described as a conditional RV x−X|X<x which indicates the time elapsed from the failure of a unit provided that its lifetime is less than or equal to time *x* (see, Nanda et al. [27]). For the Type I HLOW-G family, the *r*th order moment of the RRLT can be derived as

(24) $$\varpi_{r}^{*}\left(x\right)=E\left[{\left(x-X\right)}^{r}|X\leq x\right]=\frac{1}{F_{\mathrm{Type}\;I\;\mathrm{HLOW}\text{-}G}\left(x,\lambda ,\beta ,\varphi \right)}\sum_{j,k=0}^{\infty }\varkappa_{{}_{j,k}}^{*}\Psi_{\left[\beta (j+1)+k\right]}^{**r}\left(x\right),$$

where $\Psi_{\left[\beta (j+1)+k\right]}^{**r}$$\left(x\right)=\int_{0}^{x}y^{r}$ $\pi_{\left(\beta (j+1)+k\right)}\left(y\right)dy$. Putting r=1 in Equation (24), we can obtain the mean RRLT (MRRLT) at age *t*.

### 3.6. Rényi Entropy (RiEy)

In general, entropy belongs to statistical mechanics, more specifically microscopic variables. The RiEy is useful in many fields such as statistical inference, problems identification in statistics, communication, physics, econometrics and pattern recognition in computer science. It is a important measure of the variation of uncertainty and complexity (see, Rényi [28]). If the RV *X* has the Type I HLOW-G family, then the RiEy can be defined as (ρ>0,ρ≠1)

(25) $$\begin{array}{cc} I_{R}\left(\rho \right) & =\frac{1}{1-\rho }\log\left[\int_{-\infty }^{\infty }f_{\mathrm{Type}\;I\;\mathrm{HLOW}\text{-}G}^{\rho }\left(x,\lambda ,\beta ,\varphi \right)dx\right]\\ \; & =\frac{1}{1-\rho }\log\left[\sum_{j,k=0}^{\infty }\Delta_{{}_{j,k}}\int_{-\infty }^{\infty }\;g{\left(x;\varphi \right)}^{\rho }\;G{\left(x;\varphi \right)}^{\beta (\rho +j)+k-\rho }dx\right], \end{array}$$

where (ρ>0,ρ≠1) and

Δj,k=(2λβ)ρ∑i=0∞(−1)j+kλj(ρ+i)jj!−2ρi−β(ρ+j)−ρ)k.

Shannon entropy (SEy) can be listed as a special case of RiEy when ρ tends to 1, where SEy $=-E\left[\log f(x;\lambda ,\beta ,\varphi )\right].$ Furthermore, many authors proposed various types of entropy with some applications, for instance, Di Crescenzo and Longobardi [29] studied the characteristics of past entropy which represents the entropy of the inactivity time of a system (t−X|X<t). Baratpour et al. [30] proposed entropy properties of record statistics. Sunoj et al. [31] introduced the interval entropy which is the use of SEy for doubly truncated random variables. It plays a significant role in studying the various aspects of a system when it fails between two time points. Another entropy concept has been reported in the statistical literature, for example, Havrda and Charvát [32], Arimoto [33], Tsallis [34], and among others. Based on the proposed family in a linear representation formula, the previous entropies can be calculated by utilizing Maple software. For ρ≠1 and ρ>0, the Havrda and Charvát (HC), Arimoto (A) and Tsallis (T) entropies can be reported as follows

$$\begin{array}{ccc} {\mathrm{HC}}_{R}\left(\rho \right) & = & \frac{1}{2^{1-\rho }-1}\left[\sum_{j,k=0}^{\infty }\Delta_{j,k}\int_{-\infty }^{\infty }{\left[g(x;\varphi )\right]}^{\rho }{\left[G(x;\varphi )\right]}^{\beta (\rho +j)+k-\rho }dx-1\right],\\ A_{R}\left(\rho \right) & = & \frac{\rho }{1-\rho }\left[{\left(\sum_{j,k=0}^{\infty }\Delta_{j,k}\int_{-\infty }^{\infty }{\left[g(x;\varphi )\right]}^{\rho }{\left[G(x;\varphi )\right]}^{\beta (\rho +j)+k-\rho }dx\right)}^{\frac{1}{\rho }}-1\right], \end{array}$$

and

$$T_{R}\left(\rho \right)=\frac{1}{\gamma -1}\left[1-\sum_{j,k=0}^{\infty }\Delta_{j,k}\int_{-\infty }^{\infty }{\left[g(x;\varphi )\right]}^{\rho }{\left[G(x;\varphi )\right]}^{\beta (\rho +j)+k-\rho }dx\right];\gamma >0,$$

respectively, where

$$\begin{array}{ccc} {\mathrm{HC}}_{R}\left(\rho \right) & = & \frac{1}{2^{1-\rho }-1}\left[\int_{-\infty }^{\infty }f_{\mathrm{Type}\;I\;\mathrm{HLOW}\text{-}G}^{\rho }\left(x;\lambda ,\beta ,\varphi \right)dx-1\right],\\ A_{R}\left(\rho \right) & = & \frac{1}{1-\rho }\left[{\left(\int_{-\infty }^{\infty }f_{\mathrm{Type}\;I\;\mathrm{HLOW}\text{-}G}^{\rho }\left(x;\lambda ,\beta ,\varphi \right)dx\right)}^{\frac{1}{\rho }}-1\right] \end{array}$$

and

$$T_{R}\left(\rho \right)=\frac{1}{\gamma -1}\left[1-\int_{-\infty }^{\infty }f_{\mathrm{Type}\;I\;\mathrm{HLOW}\text{-}G}^{\rho }\left(x;\lambda ,\beta ,\varphi \right)dx\right];\gamma >0.$$

### 3.7. Order Statistics (OrSt)

The OrSt has great importance in inference and non-parametric statistics. In view of this, here, we have derived the PDF for the *i*th OrSt of Type I HLOW-G family. Suppose $x_{1},\cdots ,x_{n}$ be a random sample (RS) from the proposed distribution family, and assume that $x_{1:n},\cdots ,x_{n:n}$ denotes the corresponding OrSt. Then, the PDF of the *i*th OrSt is given as follows

(26) fi:n(x;λ,β,φ)=n!(i−1)!(n−i)!fTypeIHLOW-G(x;λ,β,φ)FTypeIHLOW-Gi−1(x;λ,β,φ)1−FTypeIHLOW-G(x;λ,β,φ)n−i=n!(i−1)!(n−i)!∑j=0n−i(−1)jn−ijfTypeIHLOW-G(x;λ,β,φ)FTypeIHLOW-Gi+j−1(x;λ,β,φ)=n!(i−1)!(n−i)!∑j=0n−i∑m,k=0∞(−1)jn−ijΥm,k(j)π[β(m+1)+k](x;φ),

where

Υm,k(j)=2λβ∑d,h=0∞(−1)d+k+mλ(h+d)+1mm!(β(m+1)+k)−i−j−1hi+j+1d−β(m+1)−1k.

Using Equation (26), we can obtain the moments of $X_{i:n}$ for the Type I HLOW-G family.

## 4. Special Type I HLOW-G Models

### 4.1. The Type I HLOW-Fréchet (Type I HLOWFr) Distribution

Consider the CDF of the Fréchet (Fr) model with parameters $\left(a,b\right)\in {ℜ}^{+}$ (see, Table 1). Then, the CDF corresponding to the Type I HLOWFr model is

(27) $$F_{\mathrm{Type}\;I\;\mathrm{HLOWFr}}\left(x;\lambda ,\beta ,a,b\right)=\left[1-e^{-\lambda {\left(e^{{\left(\frac{a}{x}\right)}^{b}}-1\right)}^{-\beta }}\right]{\left[1+e^{-\lambda {\left(e^{{\left(\frac{a}{x}\right)}^{b}}-1\right)}^{-\beta }}\right]}^{-1};x>0.$$

**Figure 1:**
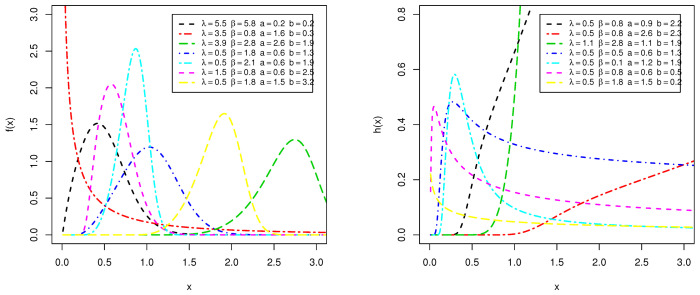
The various plots of PDF (**left panel**) and HRF (**right panel**) for Type I HLOWFr distribution.

Figure 1 illustrates various shapes of PDF and HRF of Type I HLOWFr model for different values of the parameters λ,β,a and *b*.

**Figure 2:**
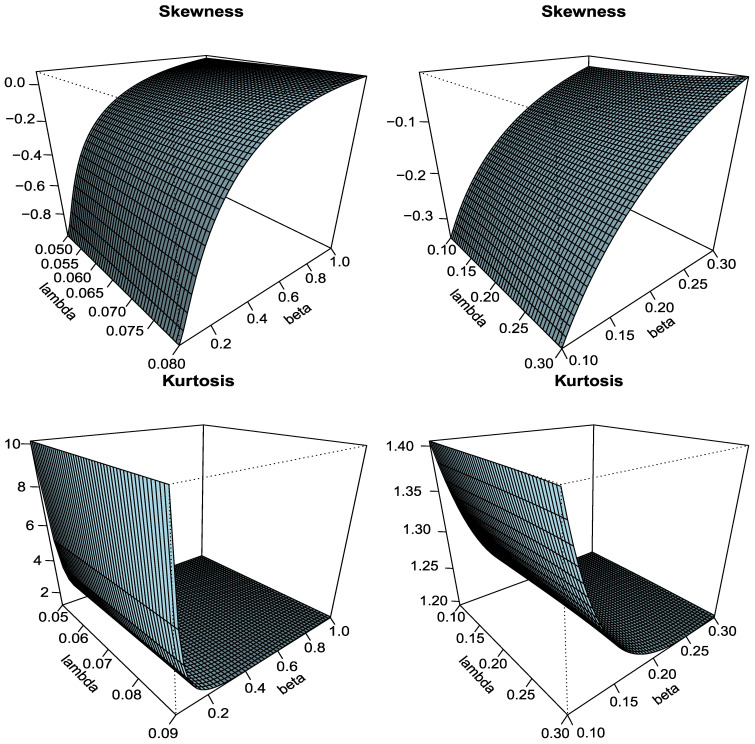
The various plots of skewness and kurtosis for Type I HLOWFr distribution.

Figure 1 shows that the PDF can be used to fit positively skewed, negatively skewed, and symmetric data sets. Moreover, the HRF can take various shapes (increasing–decreasing–unimodal). So, the Type I HLOWFr can be employed to analyze different types of data in various fields, especially, in engineering and weather forecasting areas. Based on the *r*th moment, the skewness and kurtosis of the Type I HLOWFr distribution for some particular choices of (a,b)=(0.1,3.5)“left panel” and (a,b)=(0.3,8.5) “right panel” as function of λ and β are displayed in Figure 2.

From Figure 2, we can observe that as the value of λ and β increases, the Type I HLOWFr distribution changes from negatively skewed to positively skewed, while the kurtosis of the proposed model transforms from leptokurtic to mesokurtic. Additionally, for large values of *a* and *b*, the skewness and kurtosis of the Type I HLOWFr model change rapidly. Hence, it is worth saying that the parameters λ and β greatly affect the shape of the Type I HLOWFr model.

### 4.2. The Type I HLOW-Exponential (Type I HLOWEx) Distribution

Consider the CDF of the exponential (Ex) model with parameter $a\in {ℜ}^{+}$ (see, Table 1). Then, the CDF corresponding to the Type I HLOWEx model is

(28) $$F_{\mathrm{Type}\;I\;\mathrm{HLOWEx}}\left(x;\lambda ,\beta ,a\right)=\left[1-e^{-\lambda {\left(e^{ax}-1\right)}^{\beta }}\right]{\left[1+e^{-\lambda {\left(e^{ax}-1\right)}^{\beta }}\right]}^{-1};x>0.$$

**Figure 3:**
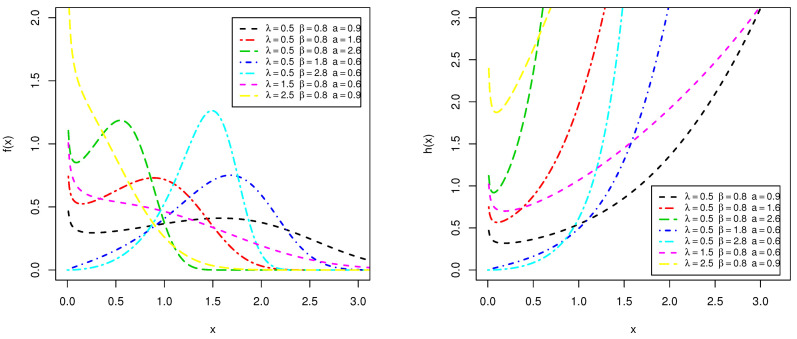
The various plots of PDF (**left panel**) and HRF (**right panel**) for Type I HLOWEx distribution.

Figure 3 depicts the PDF and HRF plots of the Type I HLOWEx distribution for different choices of the parameters λ,β and *a*. From this figure, it can be seen that the PDF of the Type I HLOWEx model can be utilized to fit negatively skewed, positively skewed and symmetric data sets. Moreover, the PDF can be uni-modal and bi-modal-shaped which can be useful in different fields, especially, in medical, weather forecasting, insurances and engineering areas. Another important advantage of Type I HLOWEx distribution is that its HRF can have different shapes (increasing–bathtub).

**Figure 4:**
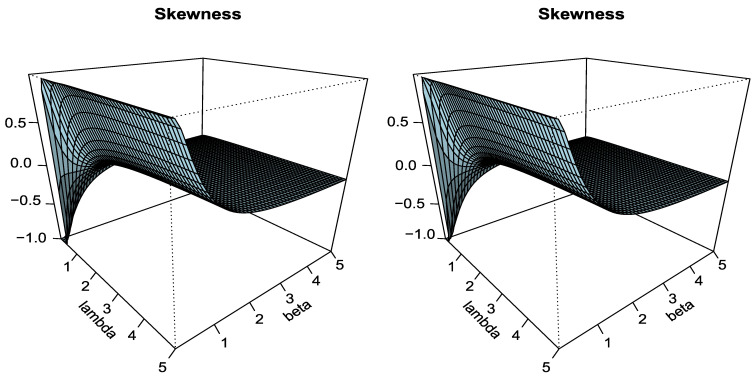

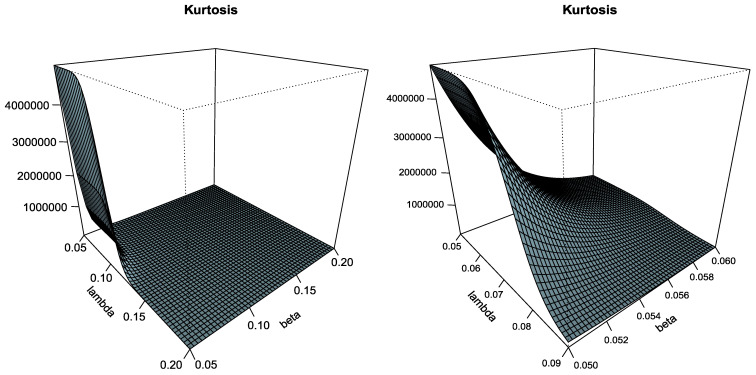
The plots of skewness and kurtosis for Type I HLOWEx distribution.

Based on the *r*th moment, the skewness and kurtosis of the Type I HLOWEx model for some choices of a=2.5“left panel” and a=3.5 “right panel” as function of λ and β are listed in Figure 4

From Figure 4, we can see that for small values of λ and β Type I HLOWEx distribution can take a negatively skewed or positively skewed shape, but as the value of β increases, it becomes symmetric. On the contrary, the kurtosis of the proposed model changes rapidly from leptokurtic to mesokurtic when the value of β and *a* increases. So, we can say that the parameters λ and β greatly affect the shape of the Type I HLOWEx model.

## 5. Different Estimation Techniques

### 5.1. Maximum Likelihood Estimation (MLE)

Suppose $x_{1},\cdots ,x_{n}$ be a RS of size *n* generated from the Type I HLOW-G family, and Θ=${\left(\lambda ,\beta ,\varphi \right)}^{T}$ be q×1 vector of unknown parameters. Then, the log-likelihood (*l*) function can be derived as

$$\begin{array}{cc} l(x;\Theta ) & =n\log\left(2\lambda \right)+n\log\left(\beta \right)+\sum_{i=1}^{n}\log g\left(x_{i};\varphi \right)+\left(\beta -1\right)\sum_{i=1}^{n}\log G\left(x_{i};\varphi \right)\\ \; & -\left(\beta +1\right)\sum_{i=1}^{n}\log\left(\bar{G}\left(x_{i};\varphi \right)\right)-\lambda \sum_{i=1}^{n}t_{i}^{\beta }-2\sum_{i=1}^{n}\log\left[1-e^{-\lambda t_{i}^{\beta }}\right], \end{array}$$

where $t_{i}=\frac{G(x_{i};\varphi )}{\bar{G}\left(x_{i};\varphi \right)}$. The components of score function $U\left(\Theta \right)={\left(U_{\lambda },U_{\beta },U_{\varphi }\right)}^{T}$ are

$$\begin{array}{cc} U_{\lambda } & =\frac{\partial l_{n}}{\partial \lambda }=\frac{n}{\lambda }-\sum_{i=1}^{n}t_{i}^{\beta }-2\sum_{i=1}^{n}\frac{t_{i}^{\beta }e^{-\lambda t_{i}^{\beta }}}{1-e^{-\lambda t_{i}^{\beta }}},\\ U_{\beta } & =\frac{\partial l_{n}}{\partial \beta }=\frac{n}{\beta }+\sum_{i=1}^{n}\log G\left(x_{i};\varphi \right)-\sum_{i=1}^{n}\log\left(\bar{G}\left(x_{i};\varphi \right)\right)\\ \; & -\lambda \sum_{i=1}^{n}t_{i}^{\beta }\log\left(t_{i}\right)-2\lambda \sum_{i=1}^{n}\frac{t_{i}^{\beta }e^{-\lambda t_{i}^{\beta }}\log\left(t_{i}\right)}{1-e^{-\lambda t_{i}^{\beta }}} \end{array}$$

and

$$\begin{array}{cc} U_{\varphi_{k}} & =\frac{\partial l_{n}}{\partial \varphi_{k}}=\sum_{i=1}^{n}\frac{g^{\prime }\left(x_{i};\varphi_{k}\right)}{g(x_{i};\varphi_{k})}+\left(\beta -1\right)\sum_{i=1}^{n}\frac{G^{\prime }\left(x_{i};\varphi_{k}\right)}{G(x_{i};\varphi_{k})}-\left(\beta +1\right)\sum_{i=1}^{n}\frac{{\bar{G}}^{\prime }\left(x_{i};\varphi_{k}\right)}{\bar{G}\left(x_{i};\varphi_{k}\right)}\\ \; & -\lambda \beta \sum_{i=1}^{n}t_{i}^{\beta -1}\left(\frac{\partial t_{i}}{\partial \varphi_{k}}\right)-2\sum_{i=1}^{n}\frac{\lambda \beta t_{i}^{\beta -1}e^{-\lambda t_{i}^{\beta }}}{1-e^{-\lambda t_{i}^{\beta }}}\left(\frac{\partial t_{i}}{\partial \varphi_{k}}\right), \end{array}$$

where $g^{\prime }\left(x_{i};\varphi_{k}\right)=\frac{\partial g(x_{i};\varphi_{k})}{\partial \delta_{k}},G^{\prime }\left(x_{i};\varphi_{k}\right)=\frac{\partial G(x_{i};\varphi_{k})}{\partial \delta_{k}},{\bar{G}}^{\prime }\left(x_{i};\varphi_{k}\right)=\frac{\partial \bar{G}\left(x_{i};\varphi_{k}\right)}{\partial \delta_{k}}$ and $\varphi_{k}$ denotes the kth item of the vector of unknown parameters. The MLEs of parameters λ,β, and $\varphi_{k}$ are extracted by solving $U_{\lambda }=U_{\beta }=U_{\varphi_{k}}=0$ and the simultaneous solution of these equations provides the MLE$\left(\hat{\Theta }\right)$. We can solve the above equations computationally through any iterative method. Since the exact distribution of the MLE is difficult to obtain, therefore by using the asymptotic distribution of the MLE, we can compute the standard errors (SEs) of the estimates. Although to obtain the MLEs and the associated SEs, we can simply use the package maxLik( ) available in the R software (R Core Development Team).

### 5.2. Simple and Weighted Least-Squares Estimators

Suppose $x_{\left(1\right)},x_{\left(2\right)},\cdots ,x_{\left(n\right)}$ be the OrSt corresponding to a RS of size *n* from the proposed distribution family. The least squares estimators (LSEs) of the family parameters, say, ${\hat{\lambda }}_{\mathrm{LS}}$, ${\hat{\beta }}_{\mathrm{LS}}$ and ${\hat{\varphi_{k}}}_{\mathrm{LS}}$ are achieved by minimizing

$$V\left(\lambda ,\beta ,\varphi \right)=\sum_{j=1}^{n}{\left[F_{\mathrm{Type}\;I\;\mathrm{HLOW}\text{-}G}\left(x_{\left(j\right)}|\lambda ,\beta ,\varphi_{k}\right)-\frac{j}{n+1}\right]}^{2},$$

with respect to λ,β, and $\varphi_{k};k=1,2,3,\cdots$. The weighted least squares estimators (WLSEs) of the Type I HLOW-G family parameters, say, ${\hat{\lambda }}_{\mathrm{WLS}}$, ${\hat{\beta }}_{\mathrm{WLS}}$ and ${\hat{\varphi_{k}}}_{\mathrm{WLS}}$ can be computed by minimizing

$$W\left(\lambda ,\beta ,\varphi \right)=\sum_{j=1}^{n}\frac{{\left(n+1\right)}^{2}\left(n+2\right)}{j\left(n-j+1\right)}{\left[F_{\mathrm{Type}\;I\;\mathrm{HLOW}\text{-}G}\left(x_{\left(j\right)}|\lambda ,\beta ,\varphi_{k}\right)-\frac{j}{n+1}\right]}^{2},$$

with respect to λ,β, and $\varphi_{k};k=1,2,3,\cdots$ Computationally, we can minimize the statistics V and W through various inbuilt functions like nls( ) function (available in the stats package of R software). Furthermore, as the exact distribution of the statistics, V and W are not easily obtainable, therefore, such functions can also be used to compute the SEs of the estimates.

### 5.3. Cramer-Von Mises Minimum Distance Estimators

Minimum distance estimators (MDEs) are those estimators that minimize the difference between theoretical and empirical CDFs. Here, we have used an eminent MDE, called the Cramér-von Mises estimator (CVME). The main advantage of this MDE is that it has less bias than other MDEs. The CVMEs of the Type I HLOW-G family parameters, say ${\hat{\lambda }}_{\mathrm{CVM}}$, ${\hat{\beta }}_{\mathrm{CVM}}$ and ${\hat{\varphi_{k}}}_{\mathrm{CVM}}\;$ are derived by minimizing

$$C\left(\lambda ,\beta ,\varphi \right)=\frac{1}{12n}+\sum_{i=1}^{n}{\left[F_{\mathrm{Type}\;I\;\mathrm{HLOW}\text{-}G}\left(x_{\left(i\right)}|\lambda ,\beta ,\varphi_{k}\right)-\frac{2i-1}{2n}\right]}^{2},$$

with respect to λ,β and $\varphi_{k};k=1,2,3,\cdots$. We can use the inbuilt functions of R software to compute the CVMEs along with the SEs of the unknown parameters.

### 5.4. Estimation through Bayesian Viewpoint

In this sub-section, we have evolved the Bayesian estimation for some particular models of the proposed family. Since, in this article, we have demonstrated Type I HLOWFr and Type I HLOWEx as special models, so we have used these distributions to draw Bayesian inferences. The main advantage of Bayesian paradigm is that it enables us to incorporate the prior knowledge with the experimental information. The prior information can be informative (non-informative) in the sense that it has a more (or less) impact on the likelihood function. The likelihood function of the Type I HLOWFr distribution can be inscribed as

L(x|λ,β,a,b)∝λnβnanbbn∏i=1nxi−bexp−β∑i=1naaxixib−λ∑i=1nexpaaxixib−1−β×∏i=1n1+exp−λexpaaxixib−1−β−21−exp−aaxixib(−β−1).

To proceed further, we have assumed independent Gamma priors for λ, β, *a*, and *b*, i.e., $\lambda ∼Gamma(c_{1},c_{2})$, $\beta ∼Gamma(d_{1},d_{2})$, $a∼Gamma(\delta_{1},\delta_{2})$, and $b∼Gamma(\nu_{1},\nu_{2})$. Here, the hyper parameters are known and non-negative and can be set to reflect the informative and non-informative beliefs about the unknown parameters. Thus, via Bayes theorem, the unnormalized joint posterior density function of (λ,β,a,b) given data can be presented as

(29) P1(λ,β,a,b|x)∝λn+c1−1exp−λc2+∑i=1nexpaaxixib−1−β×βn+d1−1exp−βd2+∑i=1naaxixib+∑i=1nlog1−exp−aaxixib×aδ1−1exp(−δ2a)∏i=1n1+exp−λexpaaxixib−1−β−21−exp−aaxixib−1×bn+ν1−1exp−bν2−nlog(a)+∑i=1nlogxi.

To obtain Bayes estimators (BEs) of the unknown parameters or any of their functions, the loss functions play a very important role. They give a measure of the financial consequences arising from an incorrect estimate of an unknown parameter. Here, we have used a well-known symmetric loss function popularized as a squared error loss (SEL) function. It takes the form as $L_{1}\left(\theta ,\hat{\theta }\right)={\left(\hat{\theta }-\theta \right)}^{2}$, where $\hat{\theta }$ is an estimate of the parameter θ. It facilitates equal weightage to negative as well as positive error. Under the SEL function, the BE of any function of parameters, Θ=(λ,β,a,b), say, ϖ(Θ) can be acquired as

(30) $${\hat{\varpi }}_{SEL}\left(\Theta |x\right)=E_{\Theta |x}\left(\varpi \left(\Theta \right)\right)=\int_{\Theta }\varpi \left(\Theta \right)P_{1}\left(\Theta |x\right)d\Theta \;\;.$$

Sometimes, in the estimation of a population parameter, positive and negative errors can have different consequences. Therefore, in such situations, we should consider a loss function that is asymmetric in nature. In view of this, here we have also used an asymmetric loss function known as generalized entropy loss (GEL) function. It can be expressed as $L_{2}\left(\theta ,\hat{\theta }\right)\propto {\left(\frac{\hat{\theta }}{\theta }\right)}^{\kappa }-\kappa \log\left(\frac{\hat{\theta }}{\theta }\right)-1,$ where the sign and magnitude of the shape parameter κ(≠0) represent the direction and degree of asymmetry, respectively. The GEL function has some important features as follows: for κ>0, GEL function gives more weightage to positive errors as compare to negative errors and vice-versa; if we set κ=−1, then BEs under GEL function becomes BEs with SEL function; for κ=1, one can obtain the BEs under entropy loss (EL) function; for κ=−2, it provides the BEs under precautionary loss (PL) function.

Under the GEL function, the BE of ϖ(Θ) can be derived as

(31) $${\hat{\varpi }}_{GEL}\left(\Theta |x\right)={\left[E_{\Theta |x}\left({\left(\varpi \left(\Theta \right)\right)}^{\left(-\kappa \right)}\right)\right]}^{-(1/\kappa )}={\left[\int_{\Theta }\varpi \left(\Theta \right)P_{1}\left(\Theta |x\right)d\Theta \right]}^{-(1/\kappa )}\;\;.$$

Due to the non-closure form of joint posterior density in Equation (29), we have used famous Monte Carlo Marko Chain (MCMC) methods such as Gibbs sampler (Geman and Geman [35]) and Metropolis-Hasting (M-H) algorithm (Metropolis and Ulam [36]; Hasting [37]). These techniques allow us to simulate complex posterior densities and to make sample-based inferences on the unknown parameters. For the execution of Gibbs sampler, the marginal distributions can be obtained as follows:

p14(b|a,x)∼Gamma(n+ν1,ν2−nlog(a)+∑i=1nlogxi),p12(β|a,b,x)∼Gamma(n+d1,d2+∑i=1n(aaxixi)b+∑i=1nlog(1−exp(−(aaxixi)b))),p11(λ|β,a,b,x)∼Gamma(n+c1,c2+∑i=1n(exp(aaxixi)b−1)−β),

and

p13(a|λ,β,b,x)∼aδ1−1exp(−δ2a)∏i=1n{(1+exp(−λ(exp(aaxixi)b−1)−β))−2(1−exp(−(aaxixi)b))−1}.

Thus, we can generate λ, β, and *b* from the above mentioned Gamma distributions under the Gibbs algorithm. Since it is not possible to generate the parameter *a* directly through $p_{13}\left(a|\lambda ,\beta ,b,x\right)$, therefore, we have used the M-H algorithm. Hence, to determine the BEs of unknown parameters of the model, the whole process can be described through the following hybrid algorithm:

Step 1. Initialize $\left(\lambda_{0},\beta_{0},a_{0},b_{0}\right)$ as starting guess of (λ,β,a,b).
Step 2. Set j=1.
Step 3. For given values of *n* and x, generate $b_{j}$, $\beta_{j}$, and $\lambda_{j}$ from their respective posterior densities $p_{14}\left(b|a_{j-1},x\right)$, $p_{12}\left(\beta |a_{j-1},b_{j},x\right)$, and $p_{11}\left(\lambda |\beta_{j},a_{j-1},b_{j},x\right)$.
Step 4. Generate $a_{j}$ from $p_{13}\left(a_{j}|\lambda_{j},\beta_{j},b_{j},x\right)$ using Normal transition distribution under the following steps of the M-H algorithm.
  (i) Generate a proposal point $a^{*}$ from a normal distribution with mean $a_{j-1}$ and variance $V_{a}$. Here, the variance $V_{a}$ can be chosen appropriately.
  (ii) Calculate the acceptance probability $\rho_{a}$ as
    $$\rho_{a}=\min\left[1,p_{13}\left(a^{*}|\lambda_{j},\beta_{j},b_{j},x\right)/p_{13}\left(a_{j-1}|\lambda_{j},\beta_{j},b_{j},x\right)\right].$$
  (iii) Draw a random uniform number $u_{1}$ from Uniform(0,1) distribution.
  (iv) If $u_{1}\leq \rho_{a}$, accept $a^{*}$ and record $a_{j}=a^{*}$, otherwise, sustain $a_{j}=a_{j-1}$.
Step 5. Set j=j+1.
Step 6. Rerun the steps 3-5, a large number of times say, *M* times and achieve $\lambda_{j},\beta_{j},a_{j}$, and $b_{j}$, j=1,2,⋯,M.

Thus, to ensure convergence and to avoid the effect of the initial guess, we have omitted the initial *N* draws. Then, the generated values, $\lambda_{j},\beta_{j},a_{j}$, and $b_{j}$, j=N+1,N+2,⋯,M, represent the required posterior samples, which can be utilized to draw the Bayesian conclusions about the unknown parameters. Hence, the BEs of ψ=λ,β,a, and *b* under SEL and GEL functions are respectively, given by

$${\hat{\psi }}_{SEL}=\frac{1}{M-N}\sum_{j=N+1}^{M}\psi_{j}\;\;\;\;\;\;\mathrm{and}\;\;\;\;{\hat{\varphi }}_{GEL}={\left[\frac{1}{M-N}\sum_{s=N+1}^{M}\varphi_{\left(s\right)}^{-\kappa }\right]}^{\left(-1/\kappa \right)}.$$

Here, it is notable that by putting the values of κ in the above expression of BEs under GEL function, we can obtain the BEs under PL and EL functions. Furthermore, to compute the posterior SE associated with a BE of a parameter, we can simply calculate the SE of the generated posterior sample corresponding to that parameter.

For Type I HLOWEx distribution, the likelihood function can be presented as

$$\begin{array}{c} L\left(x|\lambda ,\beta ,a\right)\propto \lambda^{n}\beta^{n}a^{n}\exp\left(a\beta \sum_{i=1}^{n}x_{i}-\lambda \sum_{i=1}^{n}{\left(\exp\left(ax_{i}\right)-1\right)}^{\beta }\right)\\ \;\;\;\;\;\;\;\;\;\;\;\;\;\;\;\;\;\;\;\;\;\;\;\;\;\;\;\;\;\;\;\;\times \prod_{i=1}^{n}\left\{{\left(1+\exp\left(-\lambda {\left(\exp\left(ax_{i}\right)-1\right)}^{\beta }\right)\right)}^{-2}{\left(1-\exp\left(-ax_{i}\right)\right)}^{\left(\beta -1\right)}\right\}. \end{array}$$

Further, to perform Bayesian analysis for Type I HLOWEx distribution, Gamma$\left(c_{3},c_{4}\right)$, Gamma$\left(d_{3},d_{4}\right)$, and Gamma$\left(\delta_{3},\delta_{4}\right)$ are used as the independent prior densities for the unknown parameters λ, β, and *a*, respectively. Thus, by celebrating Bayes theorem, the joint posterior distribution of (λ, β, *a*) given **x** is

(32) $$\begin{array}{c} P_{2}\left(\lambda ,\beta ,a|x\right)\propto \lambda^{n+c_{3}-1}\exp\left(-\lambda \left(c_{4}+\sum_{i=1}^{n}{\left(\exp\left(ax_{i}\right)-1\right)}^{\beta }\right)\right)\\ \;\;\;\;\;\;\;\;\;\;\;\;\;\;\;\;\;\;\;\;\;\;\;\;\;\;\;\;\;\;\;\;\;\;\;\;\times \beta^{n+d_{3}-1}\exp\left(-\beta \left(d_{4}-a\sum_{i=1}^{n}x_{i}-\sum_{i=1}^{n}\log\left(1-\exp\left(-ax_{i}\right)\right)\right)\right)\\ \;\;\;\;\;\;\;\;\;\;\;\;\;\;\;\;\;\;\;\;\;\;\;\;\;\;\;\;\;\;\;\;\;\;\;\;\times a^{n+\delta_{3}-1}\exp\left(-\delta_{4}a\right)\prod_{i=1}^{n}\left\{{\left(1+\exp\left(-\lambda {\left(\exp\left(ax_{i}\right)-1\right)}^{\beta }\right)\right)}^{-2}{\left(1-\exp\left(-ax_{i}\right)\right)}^{-1}\right\}. \end{array}$$

Thus, to achieve the BEs of λ, β, and *a* with SEL and GEL functions, the expectations of the form (30) and (31) are difficult to obtain under the joint posterior distribution in Equation (32). Therefore, by using the same hybrid algorithm as we did earlier in the Bayesian estimation of the Type I HLOWFr model, we can construct the BEs of the unknown parameters of the Type I HLOWFr distribution. For this purpose, one can obtain the marginal densities as:

$$\begin{array}{c} p_{22}\left(\beta |a,x\right)∼Gamma(n+d_{3},d_{4}-a\sum_{i=1}^{n}x_{i}-\sum_{i=1}^{n}\log(1-\exp\left(-ax_{i}\right))),\\ p_{21}\left(\lambda |\beta ,a,x\right)∼Gamma(n+c_{3},c_{4}+\sum_{i=1}^{n}{\left(\exp\left(ax_{i}\right)-1\right)}^{\beta }),\mathrm{and}\\ p_{23}\left(a|\lambda ,\beta ,x\right)∼a^{n+\delta_{3}-1}\exp\left(-\delta_{4}a\right)\prod_{i=1}^{n}\left({\left(1+\exp\left(-\lambda {\left(\exp\left(ax_{i}\right)-1\right)}^{\beta }\right)\right)}^{-2}{\left(1-\exp\left(-ax_{i}\right)\right)}^{-1}\right). \end{array}$$

## 6. The Monte Carlo Simulation Study

**Figure 5:**
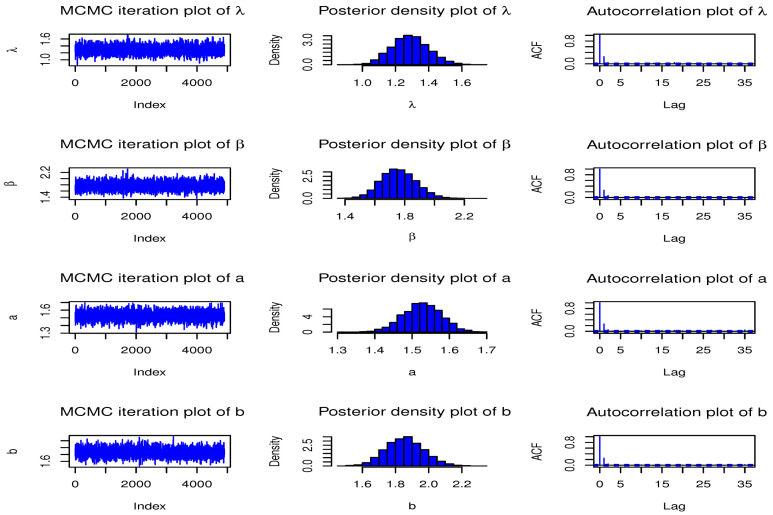
MCMC diagnostic plots for the parameters of Type I HLOWFr(λ,β,a,b)=(1.3,1.8,1.5,1.9) distribution.

**Figure 6:**
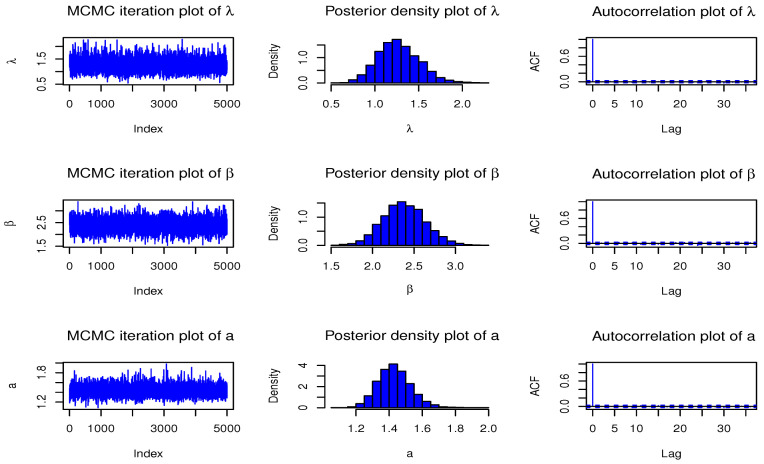
MCMC diagnostic plots for the parameters of Type I HLOWEx(λ,β,a)=(1.3,2.3,1.5) distribution.

**Figure 7:**
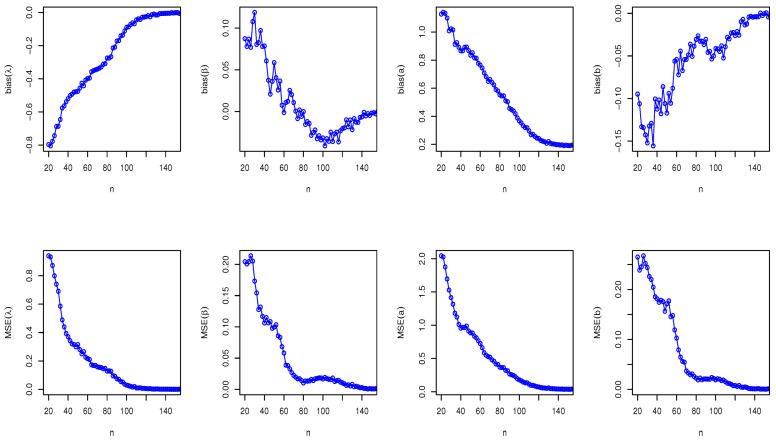
The AB and MSE of the Type I HLOWFr(λ,β,a,b)=(1.3,1.8,1.5,1.9) based on MLE method.

**Figure 8:**
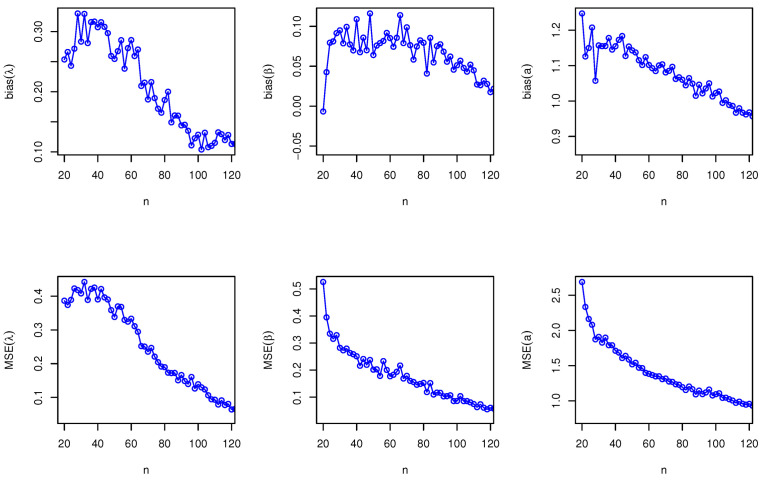
The AB and MSE of the Type I HLOWEx(λ,β,a)=(1.3,2.3,1.5) based on MLE method.

**Figure 9:**
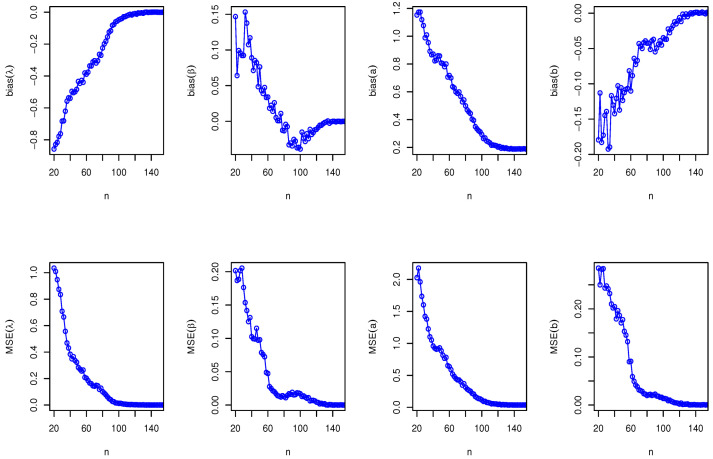
The AB and MSE of the Type I HLOWFr(λ,β,a,b)=(1.3,1.8,1.5,1.9) based on LSE method.

**Figure 10:**
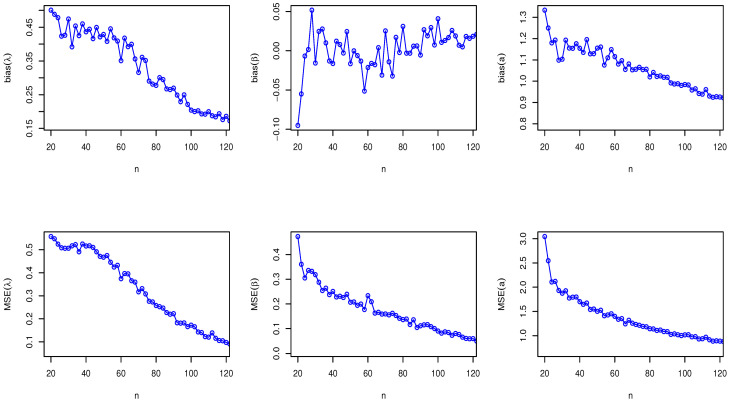
The AB and MSE of the Type I HLOWEx(λ,β,a)=(1.3,2.3,1.5) based on LSE method.

**Figure 11:**
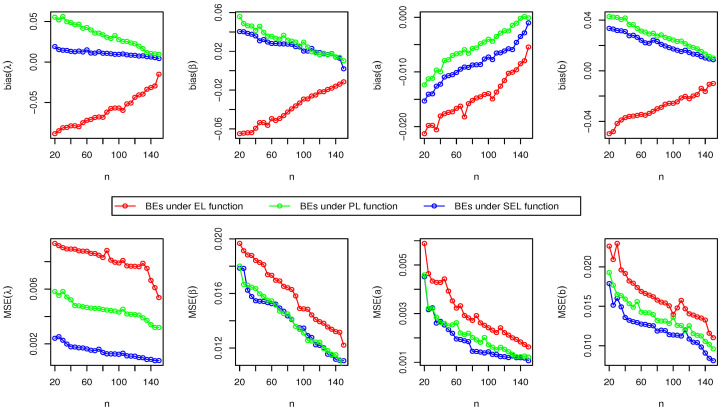
The AB and MSE of the Type I HLOWFr(λ,β,a,b)=(1.3,1.8,1.5,1.9) based on BE with IP method.

**Figure 12:**
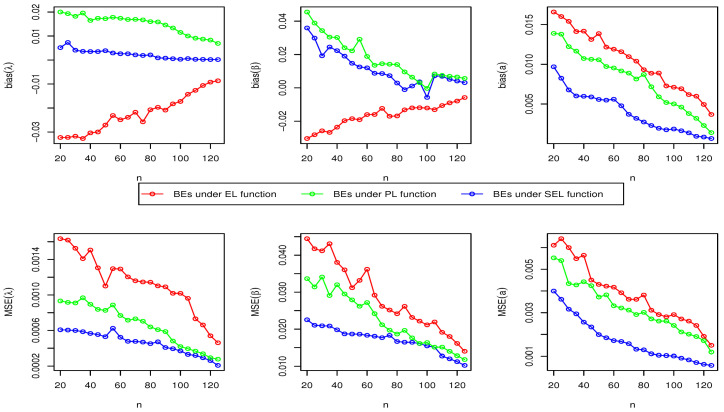
The AB and MSE of the Type I HLOWEx(λ,β,a)=(1.3,2.3,1.5) based on BE with IP method.

**Figure 13:**
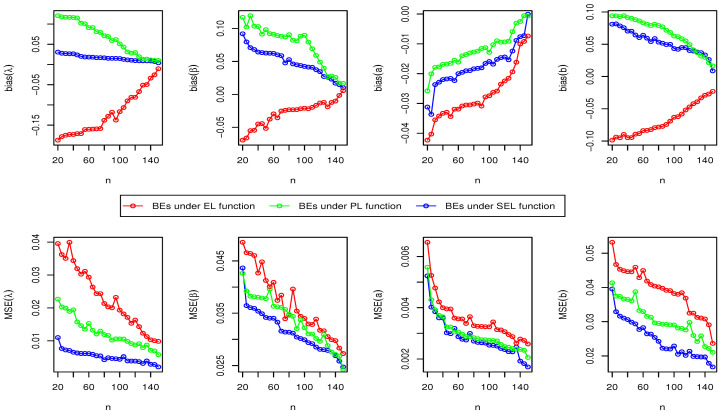
The AB and MSE of the Type I HLOWFr(λ,β,a,b)=(1.3,1.8,1.5,1.9) based on BE with NIP method.

**Figure 14:**
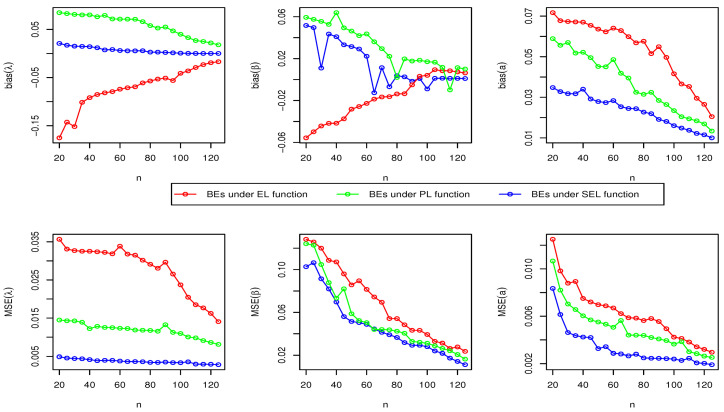
The AB and MSE of the Type I HLOWEx(λ,β,a)=(1.3,2.3,1.5) based on BE with NIP method.

Here, we have conducted a Monte Carlo simulation study to compare the behaviour of the different estimation techniques (MLEs, LSEs, WLSEs, CVMEs, and BEs) with respect to sample size *n*. In this numerical analysis, the estimation of the unknown parameters of the Type I HLOWFr and Type I HLOWEx distributions are considered. We have used R software to generate the samples and to compute the various estimates. For this purpose, we have proceeded through the following steps:

1. We have drawn 1000 samples of size n=20,25,30,⋯,150 from Type I HLOWFr (λ,β,a,b)=(1.3,1.8,1.5,1.9) and Type I HLOWEx(λ,β,a)=(1.3,2.3,1.5), respectively, through the R software.
2. We have calculated the MLEs, LSEs, WLSEs, CVMEs, and BEs for each of the 1000 samples, say ${\hat{\lambda }}_{j},{\hat{\beta }}_{j},{\hat{a}}_{j}$ and ${\hat{b}}_{j}$ for j=1,2,⋯,1000.
  It is worth noting that in the case of Bayesian estimation, we have obtained parameter estimates of Type I HLOWFr and Type I HLOWEx distributions under two types of priors viz, informative prior (IP) and non-informative prior (NIP) with SEL, PL and EL functions. For Gamma IPs, we have determined the values of hyperparameters in such a way that the expectation of the corresponding prior density of each unknown parameter is equal to the true parameter value with variance 0.4. Whereas, for NIP, with a variance as large as 2.5, we have fixed hyperparameters similar to the case of IPs. In this estimation paradigm, we have generated 55,000 MCMC draws for the parameters of Type I HLOWFr and Type I HLOWEx distribution and to ensure convergence of the chains, we have excluded first 5000 samples as burn-in period. Furthermore, to avoid the auto-correlation between the successive draws, we have stored every 10th observation. We have observed the convergence of the chains through the various MCMC diagnostic plots in Figure 5 and Figure 6. The convergence diagnostics is also performed through Geweke’s [38] criterion with a 95% credibility level. Finally, using these posterior samples, we have calculated the BEs for the unknown parameters of the Type I HLOWFr and Type I HLOWEx distributions.
3. We have calculated the average biases (ABs) and mean-squared errors (MSEs) for ψ=λ,β,a, and *b*, through the following formulas
  $$\mathrm{AB}=\frac{1}{1000}\sum_{j=1}^{1000}\left({\hat{\psi }}_{j}-\psi \right)\;\;\;\;\mathrm{and}\;\;\;\;\mathrm{MSE}=\frac{1}{1000}\sum_{j=1}^{1000}{\left({\hat{\psi }}_{j}-\psi \right)}^{2}.$$
4. The empirical results are given in Figure 7, Figure 8, Figure 9, Figure 10, Figure 11, Figure 12, Figure 13 and Figure 14, respectively. In classical scenario, the simulation study is performed only for both MLE and LSE methods because the LSE, WLSE and CVME methods gave almost the same results.

From Figure 7, Figure 8, Figure 9, Figure 10, Figure 11, Figure 12, Figure 13 and Figure 14, We come to the following important conclusions:

1. As the value of *n* increases, the magnitude of the bias decreases towards zero.
2. The MSEs of all estimators decrease when we increase the value of the sample size. This finding supports the first-order asymptotic theory.
3. In classical estimation of Type I HLOWFr model with small and moderate sample size, λ and *b* are negatively biased whereas the parameters β and *a* are positively biased. On the contrary, in Bayesian framework (with IPs or NIPs) under SEL and PL functions only the parameter *a* is underestimated and rest of the parameters are over estimated. Also, all the unknown parameters are underestimated when EL function is considered in the Bayesian analysis with IPs or NIPs.
4. For the Type I HLOWEx distribution, in the classical and Bayesian estimation with SEL and PL functions, all parameters are positively biased, whereas the BEs under EL function are negatively biased except for the BEs of the parameter *a*.
5. From the above simulation, we have observed that with respect to the sample size *n*, estimation of Type I HLOWEx parameters is more sensitive than Type I HLOWFr parameters for all estimation techniques.
6. In view of MSEs, clearly, MLE, LSE, WLSE, CVME, and BE (under IPs and NIPs) techniques perform satisfactorily in the estimation of Type I HLOWFr and Type I HLOWEx parameters. However, with respect to MSE, BEs (along with IPs and NIPs) dominates all other rival estimation procedures.
7. Particularly, in the Bayesian estimation of Type I HLOWFr and Type I HLOWEx distributions, the BEs (with NIPs and IPs) under the SEL function have outperformed the BEs (with NIPs and IPs) under PL and EL functions.

## 7. Applications

In this segment, we have demonstrated the empirical significance of the Type I HLOWFr and Type I HLOWEx models using two applications of real data. We have compared the fitted models through various measures of goodness of fit (GOF) such as the negative maximized log-likelihood (-logL), Akaike information criterion (AkIC), correct Akaike information criterion (CAkIC), Bayesian information criterion (BsIC), Hannan-Quinn information criterion (HQIC), Cramér-von Mises (CvM), Anderson-Darling (AD) statistics and Kolmogorov Smirnov (KS) statistic with the associated *p*-value.

### 7.1. Data Set I: Aluminum Coupons (AmCs)

The first data set consists the fatigue time of 101 6061-T6 AmCs cut parallel to the direction of rolling and oscillated at 18 cycles per second (cps), see Birnbaum and Saunders [39]. We have used this data to show the fitting capability of the Type I HLOWFr model relative to some other competing models such as Topp-Leone Fr (ToLFr), transmuted Fr (TrFr), exponentiated TrFr (ETrFr), Gumble Fr (GuFr), type I generalized Fr (TIGFr), exponentiated Fr (EFr) and Fr distributions. Table 2 reports the MLEs with their corresponding SEs, KS and *p*-value for AmCs data, whereas the GOF statistics are listed in Table 3.

**Figure 15:**
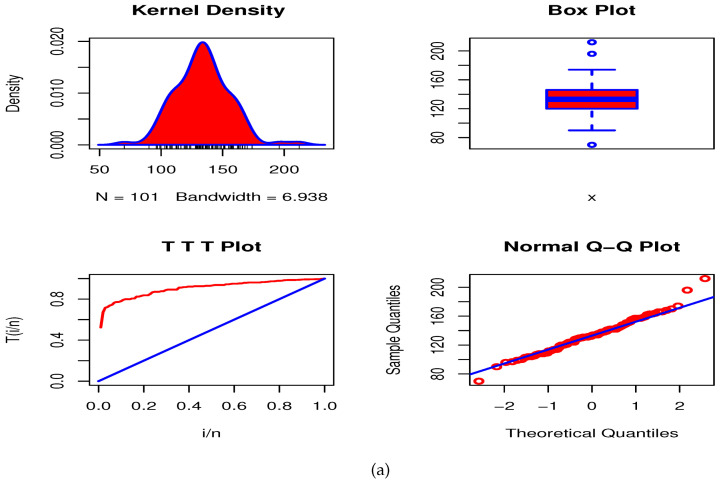

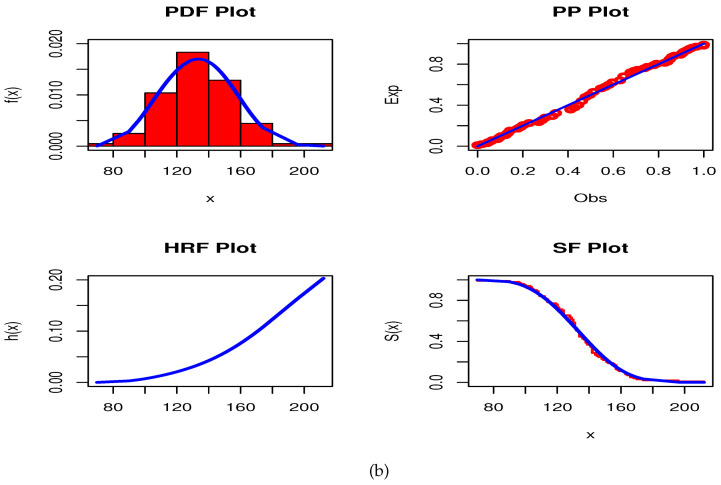
Plots for AmCs data based on the Type I HLOWFr model.

From Table 2 and Table 3, we can clearly see that the Type I HLOWFr model has the lowest values among -logL, AkIC, CAkIC, BsIC, HQIC, AD, CvM and KS. Further, the Type I HLOWFr distribution has the largest *p*-value compared to other rival distributions. Hence, the Type I HLOWFr model yields a superior fit to the AmCs data set than other fitted models. Figure 15a shows the Kernel density, box, TTT, and Normal quantile-quantile (Q-Q) plots, whereas the Figure 15b depicts the fitted PDF, probability-probability (PP), estimated HRF, and estimated survival function (SF) plots of the Type I HLOWFr model for the AmCs data. These figures support our finding obtained from Table 2 and Table 3. Also, the TTT plot in Figure 15a reveals that the data set I has an increasing failure rate, and consequently, the Type I HLOWFr model can be used to analyze this data (see, the estimated HRF in Figure 15b).

Herein, the various approaches of estimation proposed in Section 5 have utilized to estimate the unknown parameters of the Type I HLOWFr distribution. Here, it is notable that in the case of BEs, since we have no prior knowledge about the parameters of mentioned distribution, so we have used NIPs to estimate them. Table 4 lists the KS statistic and its *p*-value for these methods to get the best estimator for the AmCs data.

From Table 4, it is noted that the LSE, WLSE, CVME, and BE methods give the best estimator for this data as compared to the MLE method. Thus, we recommend using these methods for the analysis of this data. In addition, for modelling AmCs data through Type I HLOWFr distribution, according to the *p*-value, the hierarchy of the best estimation method out of these methods is:

HighlyPreferable→LessPreferable.

BE with SEL→BE with PL→BE with EL→LSE→CVME→WLSE→MLE.

**Figure 16:**
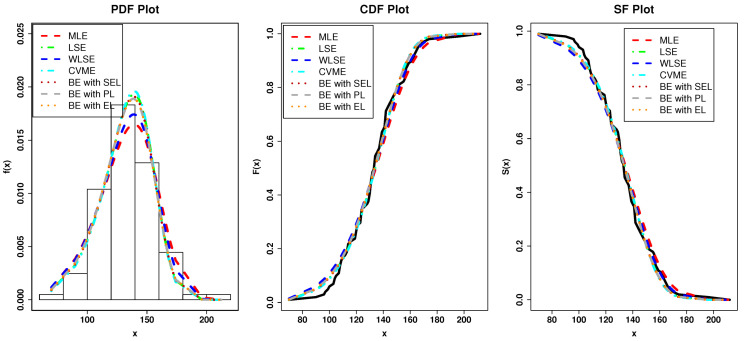
The fitted plots of the Type I HLOWFr parameters under the various method of estimation based on AmCs data.

Figure 16 shows the fitted plots based on the estimates in Table 4 which support our results.

### 7.2. Data Set II: Glass Fiber (GsFr)

This data set is reported in Smith and Naylor [40], which includes 63 observations of the strengths of 1.5 cm glass fiber, originally achieved by workers at the National Physical Laboratory, England. We have utilized this data to present the fitting capability of the Type I HLOWEx model relative to some other competing models namely, Gamma Weibull Ex (GWEx), the Extended odd Weibull Ex (ExOWEx), the modified odd Weibull Ex (MoOWEx), the odd flexible Weibull Ex (OFWEx), Topp-Leone Ex (ToLEx), odd Weibull Ex (OWEx), odd Burr-X Ex (OBuXEx), Kumaraswamy Ex (KuEx), odd log-logistic Ex (OlogLEx), odd Chen Ex (OChEx), Gumbel Ex (GuEx), exponentiated Ex (EEx), and Ex distributions. Table 5 reports the MLEs with their corresponding SEs, KS and *p*-value for GsFr data, whereas the GOF statistics can be viewed in Table 6.

**Figure 17:**
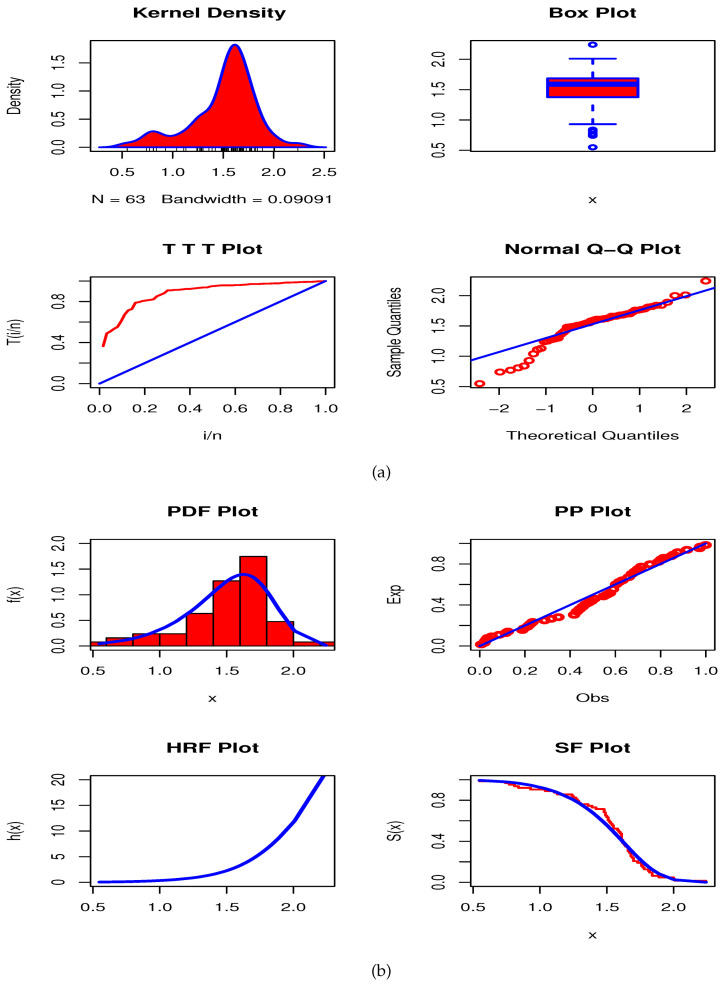
Plots for GsFr data based on the Type I HLOWEx model.

Table 5 and Table 6 reveal that the Type I HLOWEx model performs best among all fitted models. Figure 17a portrays the Kernel density, box, TTT, and Normal Q-Q plots, whereas the Figure 17b contains the fitted PDF, PP, estimated HRF, and estimated SF plots of the Type I HLOWEx distribution for the GsFr data. From these figures, we arrive at the same conclusions as we are observed from Table 5 and Table 6. Furthermore, the TTT plot in Figure 17a shows that the data set II has an increasing failure rate, and consequently, the Type I HLOWEx model can be used to analyze this data (see, the estimated HRF in Figure 17b).

Table 7 reports the KS statistic and the associated *p*-value for various estimation methods under GsFr data. It is worth noting here that to extract the BEs for data set II, we have used NIPs for the unknown parameters of the Type I HLOWEx distribution.

From Table 7, it is observed that the LSE, WLSE, CVME, and BE methods give the best estimator for this data as compared to the MLE method. Thus, we recommend using these methods for the analysis of the GsFr data. In addition, for modelling GsFr data through Type I HLOWEx distribution, according to the *p*-value, the hierarchy of the best estimation method out of these methods is:

HighlyPreferable→LessPreferable.

BE with SEL→BE with PL→BE with EL→CVME→LSE→WLSE→MLE.

**Figure 18:**
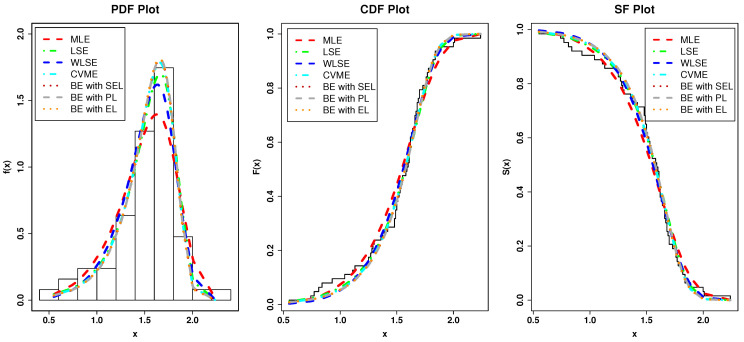
The fitted plots of the Type I HLOWEx parameters under the various method of estimation based on GsFr data.

Figure 18 shows the fitted plots based on the estimates in Table 7 which support our results.

### 7.3. Some Descriptive Statistics for Data Sets I and II

Table 8 lists some descriptive statistics for data sets I and II based on BEs under SEL function. Here, it is notable that with the help of Maple software we have obtained various measures of central tendency and dispersion for real data sets.

It is clear that empirical and theoretical measures are approximately equal. This implies that the proposed models are appropriate to analyze data sets I and II.

## 8. Conclusions

In the above study, we have proposed a new generator of univariate continuous distributions, called Type I HLOW-G family. Its various statistical features have been derived. The special sub-models of the Type I HLOW-G family are capable of modelling positively skewed, negatively skewed, and symmetric data sets. Moreover, these sub models provide a wide variation in the shape of the HRF, including decreasing, increasing, unimodal, and bathtub shapes, and consequently the generated distributions can be used in modelling several types of data. Two particular models of the proposed family have been extensively studied, in the so-called Type I HLOWFr and Type I HLOWEx distributions. The model parameters have been estimated using five different estimation methods, namely, MLE, LSE, WLSE, CVME, and BE. In the Bayesian paradigm, we have obtained the Bayes estimates under informative and non-informative priors with squared error, precautionary and entropy loss functions. The two special cases of the Type I HLOW-G class have been applied to two real-life data sets to illuminate the fitting superiority of the Type I HLOW-G family over other existing rival models. From these numerical examples, we can also say that the last four methods of estimation provide good estimators compared to MLE, especially in this family. Finally, we can conclude that the Type I HLOW-G family would be a better alternative to other existing continuous distributions for modelling real data generated from different areas.

## Figures and Tables

**Table 1:** Some special models of Type I HLOW-G family.

G(x;φ)	$\frac{G(x;\varphi )}{\bar{G}\left(x;\varphi \right)}$	Reduced Distribution	φ
$1-e^{-ax}$	$e^{ax}-1$	Type I HLOW-exponential	*a*
$1-e^{-\frac{a}{2}x^{2}}$	$e^{\frac{a}{2}x^{2}}-1$	Type I HLOW-Rayleigh	*a*
$1-\left(1+\frac{ax}{a+1}\right)e^{-ax}$	${\left(1+\frac{ax}{a+1}\right)}^{-1}e^{ax}-1$	Type I HLOW-Lindely	*a*
$1-e^{-{\left(\frac{x}{b}\right)}^{a}}$	$e^{{\left(\frac{x}{b}\right)}^{a}}-1$	Type I HLOW-Weibull	(a,b)
$e^{-{\left(\frac{a}{x}\right)}^{b}}$	${\left(e^{{\left(\frac{a}{x}\right)}^{b}}-1\right)}^{-1}$	Type I HLOW-Fréchet	(a,b)
$1-{\left(1+\frac{x}{b}\right)}^{-a}$	${\left(1+\frac{x}{b}\right)}^{a}-1$	Type I HLOW-Lomax	(a,b)

**Table 2:** The MLEs with their (SEs) and KS with its *p*-value for AmCs data.

Model	$\;\;\;\;\;\hat{\lambda }\;\;\;\;\;\;$	$\;\;\;\;\hat{\beta }\;\;\;\;\;$	$\hat{a}$	$\hat{b}$	KS	*p*-Value
**Type I HLOWFr**	0.0804	3.1059	65.4987	1.4272	0.0891	0.3987
	(0.0014)	(0.0145)	(0.1753)	(0.4523)	−	−
**ToLFr**	35.0775	0.7832	59.6911	4.0886	0.1214	0.1017
	(2.0369)	(0.5881)	(2.0365)	(0.4291)	−	−
**TrFr**	0.9991	−	136.9523	3.9802	0.1208	0.1049
	(0.1074)	−	(5.7146)	(0.8631)	−	−
**ETrFr**	−0.9979	0.2594	136.9564	5.5829	0.1235	0.0918
	(0.0034)	(0.1286)	(12.3828)	(0.3745)	−	−
**GuFr**	1.9688	0.02939	3.4574	0.1072	0.1351	0.0500
	(0.0041)	(0.0032)	(0.0147)	(0.0079)	−	−
**TIGFr**	16648.9937	74.4737	7.5315	5.0567	0.1328	0.0567
	(7108.4949)	(275.7257)	(5.6109)	(0.3249)	−	−
**EFr**	−	73.2216	51.6791	5.0575	0.1329	0.0563
	−	(0.1473)	(0.1421)	(0.3252)	−	−
**Fr**	−	−	120.7822	5.0575	0.1329	0.0563
	−	−	(2.5251)	(0.3252)	−	−

**Table 3:** The GOF Statistics for the AmCs data.

Model	−logL	AkIC	CAkIC	BsIC	HQIC	AD	CvM	DF
**Type I HLOWFr**	461.1651	930.3302	930.7469	940.7907	934.5649	0.8685	0.1369	4
**ToLFr**	466.3545	940.7090	941.1256	951.1695	944.9437	1.5426	0.2704	4
**TrFr**	466.4059	938.8118	939.0592	946.6571	941.9878	1.5649	0.2750	3
**ETrFr**	468.9391	945.8782	946.2949	956.3387	950.1129	1.9234	0.3470	4
**GuFr**	475.7321	959.4625	959.8792	969.9230	963.6972	2.5578	0.4431	4
**TIGFr**	475.1911	958.3821	958.7988	968.8426	962.6168	2.4967	0.4329	4
**EFr**	475.1857	956.3714	956.6188	964.2168	959.5474	2.4971	0.4330	3
**Fr**	475.1857	954.3714	954.4939	959.6017	956.4888	2.4971	0.4330	2

**Table 4:** Different estimation techniques for the Type I HLOWFr parameters with KS, (SE) and *p*-values for the AmCs data.

Method ↓Estimator→	$\hat{\lambda }$	$\hat{\beta }$	$\hat{a}$	$\hat{b}$	KS	*p*-Value
**LSE**	0.4772(0.1265)	17.3435(0.0597)	27.8989(0.0867)	0.2557(0.2540)	0.0608	0.8487
**WLSE**	0.5212(0.1169)	16.0827(0.0308)	26.8382(0.0871)	0.2491(0.9537)	0.0705	0.6966
**CVME**	0.4723(0.1025)	17.5159(0.0471)	28.1555(0.0497)	0.2572(0.5674)	0.0633	0.8132
**BE with SEL function**	0.4696(0.0489)	17.1055(0.0226)	27.7908(0.0392)	0.2562(0.0392)	0.0602	0.8574
**BE with PL function**	0.4656(0.0743)	17.0156(0.0281)	27.3808(0.0441)	0.2544(0.0429)	0.0605	0.8523
**BE with EL function**	0.4659(0.0763)	17.0198(0.0293)	27.3818(0.0469)	0.2544(0.0431)	0.0608	0.8492

**Table 5:** The MLEs with their (SEs) and KS with its *p*-value for the GsFr data.

Model	$\;\;\;\;\;\hat{\lambda }\;\;\;\;\;\;$	$\;\;\;\;\hat{\beta }\;\;\;\;\;$	$\hat{a}$	$\hat{b}$	KS	*p*-Value
**Type I HLOWEx**	0.4016	3.3597	0.5530	−	0.1317	0.2240
	(2.4146)	(1.6865)	(0.7175)	−	−	−
**GWEx**	0.0292	0.6772	3.8165	1.9018	0.1318	0.2236
	(0.0656)	(0.6310)	(3.2469)	(1.2361)	−	−
**ExOWEx**	4.4343	0.0667	0.4272	−	0.1443	0.1452
	(0.6423)	(0.1572)	(0.0121)	−	−	−
**MoOWEx**	0.0495	3.2822	0.9107	−	0.1357	0.1966
	(0.1499)	(1.5824)	(0.6762)	−	−	−
**OFWEx**	3.2814	1.4226	0.3114	−	0.1659	0.0625
	(8.5346)	(2.5169)	(0.5322)	−	−	−
**ToLEx**	55.0916	0.4353	2.1965	−	0.2253	0.0033
	(48.9007)	(0.3207)	(0.2828)	−	−	−
**OWEx**	0.0148	2.8843	1.0155	−	0.1362	0.1928
	(0.0185)	(0.7872)	(0.3879)	−	−	−
**OBuXEx**	3.0166	−	0.5428	−	0.1894	0.0218
	(0.5366)	−	(0.02004)	−	−	−
**KuEx**	$6.08\times 10^{7}$	$7.64\times 10^{5}$	0.0701	−	0.1549	0.0973
	(0.7092)	$\left(1.98\times 10^{4}\right)$	$\left(1.89\times 10^{-2}\right)$	−	−	−
**OLogLEx**	5.9148	−	0.4533	−	0.1480	0.1264
	(0.6555)	−	(0.0118)	−	−	−
**OChEx**	45.5507	4.7936	0.2273	−	0.1469	0.1314
	(189.9452)	(0.9314)	(0.1366)	−	−	−
**GuEx**	34.5036	4.3097	11.4467	−	0.2223	0.0039
	(9.9112)	(0.4542)	(0.6979)	−	−	−
**EEx**	−	31.3380	2.6110	−	0.2289	0.0027
	−	(9.5154)	(0.2379)	−	−	−
**Ex**	−	−	0.6636	−	0.4179	0.0000
	−	−	(0.0836)	−	−	−

**Table 6:** The GOF Statistics for the GsFr data.

Model	−logL	AkIC	CAkIC	BsIC	HQIC	AD	CvM	DF
**Type I HLOWEx**	13.5276	33.0552	33.4620	39.4846	35.5839	0.8581	0.1529	3
**GWEx**	14.1821	36.3643	37.0539	44.9368	39.7359	0.8938	0.1586	4
**ExOWEx**	14.4938	34.9875	35.3943	41.4169	37.5162	1.1438	0.2079	3
**MoOWEx**	14.3855	34.7709	35.1777	41.2004	37.2997	0.9762	0.1736	3
**OFWEx**	16.0906	38.1812	38.5879	44.6106	40.7099	1.5051	0.2749	3
**ToLEx**	26.0928	58.1856	58.5924	64.6149	60.7143	3.4699	0.6342	3
**OWEx**	14.3900	34.7800	35.1868	41.2094	37.3087	0.9848	0.1754	3
**OBuXEx**	17.8984	39.7969	39.9969	44.0832	41.4827	1.9488	0.3559	2
**KuEx**	15.3599	36.7199	37.1267	43.1493	39.2486	1.3578	0.2477	3
**OLogLEx**	20.4244	44.8487	45.0487	49.1349	46.5345	2.3459	0.4251	2
**OChEx**	14.8415	35.6830	36.0898	42.1124	38.2117	1.1938	0.2163	3
**GuEx**	30.5286	67.0573	67.4640	73.4867	69.5859	4.1291	0.7554	3
**EEx**	31.3937	66.7874	66.9874	71.0737	68.4732	4.2977	0.7886	2
**Ex**	88.8369	179.6739	179.7395	181.8170	180.5168	3.1379	0.5727	1

**Table 7:** Different estimation techniques for the Type I HLOWEx parameters with KS, (SE) and *p*-values for the GsFr data.

Method ↓ Estimator →	$\hat{\lambda }$	$\hat{\beta }$	$\hat{a}$	KS	*p*-Value
**LSE**	0.0028(0.0147)	0.7843(0.1397)	4.8391(0.0917)	0.0835	0.7714
**WLSE**	0.1689(0.1294)	3.7036(0.0199)	0.6280(0.0199)	0.1036	0.5080
**CVME**	0.0021(0.0437)	0.9555(0.0497)	4.1670(0.1257)	0.0768	0.8516
**BE with SEL function**	0.0019(0.0013)	0.9711(0.0121)	4.1595(0.0129)	0.0719	0.8998
**BE with PL function**	0.0019(0.0067)	0.9706(0.0149)	4.1575(0.0157)	0.0749	0.8708
**BE with EL function**	0.0019(0.0093)	0.9708(0.0176)	4.1681(0.0183)	0.0753	0.8663

**Table 8:** Empirical and theoretical descriptive statistics for data sets I and II.

Data Set	Measure	Mean	Variance	Skewness	Kurtosis	Index of Dispersion
I	Empirical	131.277	499.965	−0.5725	3.3745	3.8084 (over-dispersed)
	Theoretical	133.732	499.777	−0.3354	4.2608	3.7372(over-dispersed)
II	Empirical	1.5123	0.1038	−0.9456	3.9867	0.0697(under-dispersed)
	Theoretical	1.5069	0.1051	−0.9235	4.2559	0.0697(under-dispersed)

## Data Availability

The data that we have used is openly available and can be viewed from the studies of Birnbaum and Saunders [39] and Smith and Naylor [40].
